# Core functions of a financial navigation intervention: An in-depth assessment of the Lessening the Impact of Financial Toxicity (LIFT) intervention to inform adaptation and scale-up in diverse oncology care settings

**DOI:** 10.3389/frhs.2022.958831

**Published:** 2022-11-09

**Authors:** Stephanie B. Wheeler, Sarah A. Birken, Cheyenne R. Wagi, Michelle L. Manning, Mindy Gellin, Neda Padilla, Cindy Rogers, Julia Rodriguez, Caitlin B. Biddell, Carla Strom, Ronny Antonio Bell, Donald L. Rosenstein

**Affiliations:** ^1^Lineberger Comprehensive Cancer Center, University of North Carolina at Chapel Hill, Chapel Hill, NC, United States; ^2^Department of Health Policy and Management, University of North Carolina at Chapel Hill, Chapel Hill, NC, United States; ^3^Department of Implementation Science, Wake Forest University School of Medicine, Winston-Salem, NC, United States; ^4^Wake Forest Baptist Comprehensive Cancer Center, Winston-Salem, NC, United States; ^5^Department of Psychiatry, University of North Carolina at Chapel Hill, Chapel Hill, NC, United States

**Keywords:** financial toxicity, cancer, financial navigation, adaptation, implementation

## Abstract

**Background:**

Lessening the Impact of Financial Toxicity (LIFT) is an intervention designed to address financial toxicity (FT) and improve cancer care access and outcomes through financial navigation (FN). FN identifies patients at risk for FT, assesses eligibility for financial support, and develops strategies to cope with those costs. LIFT successfully reduced FT and improved care access in a preliminary study among patients with high levels of FT in a single large academic cancer center. Adapting LIFT requires distinguishing between core functions (components that are key to its implementation and effectiveness) and forms (specific activities that carry out core functions). Our objective was to complete the first stage of adaptation, identifying LIFT core functions.

**Methods:**

We reviewed LIFT's protocol and internal standard-operating procedures. We then conducted 45–90 min in-depth interviews, using Kirk's method of identifying core functions, with key LIFT staff (*N* = 8), including the principal investigators. Interviews focused on participant roles and intervention implementation. Recorded interviews were transcribed verbatim. Using ATLAS.ti and a codebook based on the Model for Adaptation Design and Impact, we coded interview transcripts. Through thematic analysis, we then identified themes related to LIFT's intervention and implementation core functions. Two report back sessions with interview participants were incorporated to further refine themes.

**Results:**

Six intervention core functions (i.e., what makes LIFT effective) and five implementation core functions (i.e., what facilitated LIFT's implementation) were identified to be sufficient to reduce FT. Intervention core functions included systematically cataloging knowledge and tracking patient-specific information related to eligibility criteria for FT relief. Repeat contacts between the financial navigator and participant created an ongoing relationship, removing common barriers to accessing resources. Implementation core functions included having engaged sites with the resources and willingness necessary to implement FN. Developing navigators' capabilities to implement LIFT—through training, an established case management system, and connections to peer navigators—were also identified as implementation core functions.

**Conclusion:**

This study adds to the growing evidence on FN by characterizing intervention and implementation core functions, a critical step toward promoting LIFT's implementation and effectiveness.

## Introduction

Cancer care in the United States is associated with substantial—and in many cases, ongoing—financial burdens that patients with cancer and their families struggle to manage. Individuals historically underserved by medical institutions (e.g., patients living in rural areas, non-English speaking patients, patients of color) experience disproportionate financial burden and poor access to cancer care (1–3). Mounting evidence has documented the extraordinary burden of out-of-pocket medical and non-medical expenses on patients, leading to an increased risk of downstream adverse consequences, such as worse health-related quality of life, care avoidance and discontinuation, bankruptcy, and mortality (4–8). Collectively, these risks have been termed financial toxicity (FT). In addition to leading to harmful consequences for patients, FT has also been shown to negatively impact caregivers and other members of the household (9, 10). Furthermore, FT may negatively impact healthcare system finances through cost-related missed appointments and uncompensated care (11, 12).

Increasing awareness of this problem has motivated the development of interventions to prevent or mitigate FT (13, 14). Although validated measures exist to identify FT (15, 16), and most NCI-designated cancer centers and National Comprehensive Cancer Network member institutions report providing some forms of financial support for their patients (17, 18), cancer centers' approaches to systematic FT identification and mitigation are highly variable and, as a result, variably effective. For cancer care providers to more effectively reach and assist patients with disproportionate financial burden, it is essential that structured interventions are developed and disseminated with those communities in mind.

One such structured intervention to address FT is financial navigation (FN). FN identifies patients at risk for or experiencing FT, educates patients about programs and services that may help address FT, directly assists patients in applying for, and receiving benefits from, existing programs and services, and tracks and manages patient needs in an ongoing manner (4, 19). Administered by a trained financial navigator, FN is designed to build capacity to address financial needs of patients with cancer and improve quality of care, while reducing duplication of effort and integrating workflows across cancer supportive care service providers. FN training typically includes education about health insurance and government structures, policies and resources and tools and techniques to assess and address financial concerns of patients (e.g., through the Association of Community Cancer Centers Financial Advocacy Bootcamp). The ACCC Financial Advocacy Bootcamp is a national, online resource provides basic information on federal financial aid programs and eligibility requirements, patient communication recommendations, and problem-solving strategies (20). FN training also typically includes orientation to the needs and care trajectories of patients with cancer (through evidence reviews, testimonials, and other mechanisms); case management skills-building and tracking; review of local, state, and national resources for financial support services and eligibility; review of relevant case management protocols (e.g., frequency and duration of case sessions, protection of privacy and confidentiality, referral processes); and access to senior FN specialists who train and mentor other financial navigators and can share personal experiences with FN.

FN interventions have been shown to decrease patient financial distress, provide material financial support, and improve revenue recovery for uncompensated care at hospitals (4, 19). To extend the benefits of FN to new care contexts and populations, such as patients living in rural areas, we must first identify the intervention core functions of FN—i.e., the features that drive its effectiveness and thus cannot be compromised. Simultaneously, we must understand the features of FN that are required for its integration into routine clinical practice (i.e., its implementation core functions) since poor implementation will compromise effectiveness. Understanding the intervention and implementation core functions of an evidence-based FN intervention will equip cancer programs with the knowledge required to adapt FN to new contexts and populations without compromising its effectiveness. To that end, our objective in this manuscript was to identify FN core functions to facilitate adaptation for implementation in diverse populations and contexts.

## Materials and methods

### Study design

We used Kirk et al.'s methods of identifying core functions for an evidence-based FN intervention—Lessening the Impact of Financial Toxicity (LIFT).

### Lessening the Impact of Financial Toxicity (LIFT) program

The LIFT program was tested at the UNC Lineberger Comprehensive Cancer Center in 2019. The initial intervention study was supported by an internal grant from the UNC Innovation Center and was approved by the UNC IRB (UNC IRB # 18-2765). The FN intervention consisted of (1) systematic identification of cancer patients at high risk for FT using the Comprehensive Score for Financial Toxicity (COST) measure (COST ≤ 22 considered high risk for FT) (15, 16); (2) connection of patients experiencing FT, or at high risk for FT, with dedicated and trained oncology financial navigators, who employed a comprehensive assessment tool to determine financial needs and one-on-one appointments to direct patients to specific financial support resources and assist with applications; and (3) routine electronic tracking and monitoring of patients' financial and health outcomes. The intervention included regular, biweekly phone or in-person check-ins for up to 6 months, or until patients reported meeting one or more of their financial goals. Outcomes of this program have been reported elsewhere. Briefly, the FN intervention was associated with a statistically significant, nearly 7-point improvement in patient-reported financial distress (measured by the COST instrument, range: 0–44), and patients viewed LIFT as acceptable, timely and highly responsive to their needs (21).

### Data collection and study procedures

**Figure 1:**
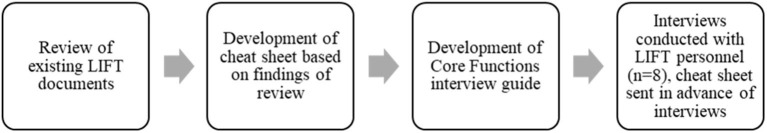
Core functions data collection sequence.

SAB and CRW led the process of identifying LIFT core functions using Kirk et al.'s theory-based method using a multi-step system (Figure 1). First, SAB and CRW reviewed the existing documents describing LIFT, including the LIFT protocol, internal standard operating procedures, and patient-facing materials. Second, SAB and CRW developed a “cheat sheet” (Appendix 1) that provided an overview of perceived core functions of the LIFT program based on the review of existing documents. This cheat sheet was sent to participants to review in advance of the interview and used during the interview to guide discussion. Third, SAB and CRW developed a semi-structured interview guide (Appendix 2) designed to enhance understanding of LIFT forms, and how those forms were thought to drive LIFT's implementation and effectiveness. The interview guide was designed based on Kirk et al.'s methods of identifying core functions, with additional questions added to fill in gaps and answer questions from the initial data collection from existing documents. Questions fell into categories of participant information, questions about accuracy and gaps of the “cheat sheet,” causal pathways, core functions of the intervention, and ways in which the intervention would need to be adapted for additional populations. Interviews were conducted by SAB and CRW with eight UNC Lineberger faculty, staff, administrators, and research team members who were involved in LIFT's development and pilot testing. Both SAB and CRW are trained in qualitative methods, have significant experience conducting qualitative research, and conducting semi-structured interviews. Participants were asked about their roles and LIFT's central implementation and programmatic activities. Additional questions focused on the aspects of the LIFT study that were specific to the site infrastructure and patient demographics for the purposes of future adaptation. Each interview was conducted in person or on Webex in 2021 and lasted 45–90 min. An interviewer's report summarizing key points, notable quotes, and overall findings was drafted at the conclusion of each interview. Digital recordings of each interview were independently transcribed verbatim.

### Analysis

Using ATLAS.ti version 22 (22), SAB and CRW then coded the existing LIFT documents and interview transcripts using the Model for Adaptation Design and Impact (23). Thematic analysis was used to understand how LIFT was integrated into clinical practice (implementation core functions) and decreased FT (intervention core functions). Themes were initially built by identifying patterns related to LIFT core functions and organizing those themes into related groups. In a thorough member-checking exercise, SAB and CRW met with interview participants to refine the themes. They first discussed an initial draft of themes with SBW and DR, LIFT developers, to refine the themes based on their experience with LIFT. CW and SAB then presented the refined themes in two subsequent meetings with other interview participants. Once interview participants had no additional feedback on the themes, CW and SAB classified themes as either implementation or intervention core functions. Intervention core functions were defined as intervention components that were necessary and sufficient in combination to achieve the intended effectiveness outcome of reduced FT. Implementation core functions were components that were necessary and sufficient in combination to achieve the intended implementation outcomes of acceptability and feasibility. We then developed a spreadsheet with this information for interview participants' final review of core functions' accuracy and comprehensiveness.

Finally, to explain the change underlying LIFT's implementation and effectiveness—i.e., the mechanism(s) thought to drive LIFT's integration into practice and reduction of FT—SAB identified relevant organization theories. Based on concepts related to power, autonomy, and control, organization theories explain how and why interventions such as LIFT are adopted, implemented, and sustained in new contexts.

## Results

Interview participants included the principal investigators who initially designed LIFT (SBW and DR), the LIFT project director (MM), the LIFT program manager (MG), two research assistants (NP and CB), and the two financial navigators engaged in the original pilot study (CR and JR).

### Intervention structure

**Figure 2:**
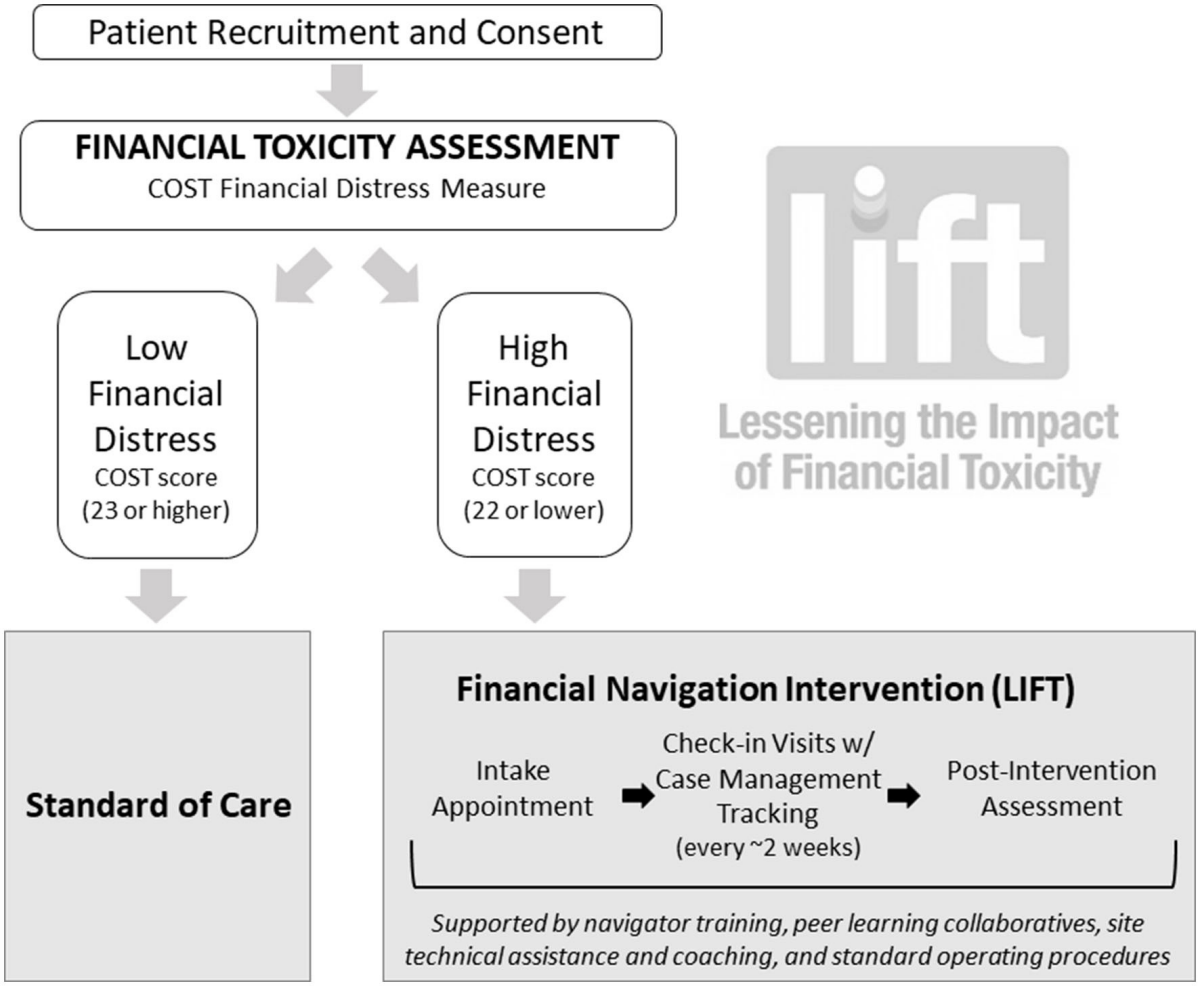
LIFT financial navigation study flow chart.

Figure 2 details the structure of the LIFT intervention. Patients referred for FN were identified through multiple channels (self-referral, provider referral, electronic health record system-facilitated referral) and typically had at least 2 visits with the financial navigator with some patients receiving more intensive, needs-dependent support. Repeated visits with the financial navigator were often important for navigators to review patients' eligibility and applications for financial support services, clarify paperwork needs, correct errors, and assist with application submission. Appointments involved one-on-one consultation with the financial navigator, who assessed patients' individual and household financial situation and financial assistance goals. Financial navigators also collected information about employment status, billing information, insurance status and other indicators used to triage patients to the appropriate financial resource(s). At the end of this initial appointment and comprehensive intake assessment, patients were provided a checklist of resources they were potentially eligible for and a list of the personal paperwork (e.g., tax forms, W-2, pay stubs) needed to apply. During the follow-up appointment(s), the financial navigator reviewed the initial intake forms, verified that the patient had the necessary paperwork and worked with the patient to complete resource applications. Patients were educated about and referred to financial resources, including but not limited to, hospital-based assistance programs, local nonprofits, foundation-provided financial support, medication assistance programs, Medicaid, Medicare, private health insurance plans, Social Security Disability Insurance and Supplemental Security Income, and legal assistance. Patients were re-contacted by the financial navigator 2–3 weeks after each FN clinic visit to assess progress toward their financial assistance goals. The intervention lasted between 2 weeks and 6 months, with an average intervention time of 4 months, depending on needs. The original FN intervention was delivered in-person by social work-trained navigators at varying ranks and levels, from senior oncology social worker to social work student trainees.

We identified six intervention core functions that were collectively necessary and sufficient to reduce FT, and five implementation core functions that drove LIFT's integration into clinical practice (see Tables 1, 2, respectively).

**Table 1:** LIFT financial navigation intervention core functions.

**LIFT intervention core functions**		
**Core function: the “what”—what makes LIFT effective?**	**Example forms: the “how”—how has LIFT accomplished the core function?**	**Example quotes**
1. Provided a systematic way of *cataloging* knowledge, structures, and programs to reduce cancer-related financial hardship	• Comprehensive intake forms and tracking	• “It was like harnessing knowledge that existed but wasn't always systematized in a way that—every encounter might've been a little bit different, and pulling all that information together and coordinating it and then, yeah, like you said, structuring, organizing it, and helping improve the process so that the process itself didn't rely on one person's institutional knowledge.”
		• As I'm thinking about what we've shared with you so far, I wonder whether we've sufficiently emphasized the importance of the screening process. We've mentioned it, but it's really key to have something systematic and objective and actionable.
2. Provided a systematic way of tracking patient information that informs eligibility for knowledge, structures, and programs to reduce cancer-related financial hardship and application status	• Comprehensive intake forms and tracking	• “Once identified, having a comprehensive intake assessment that wasn't just, “Tell me about your job,” or, “Tell me about your insurance status,” but that was driven by—almost like a checklist of, “Okay, well, let me just go through and make sure that I've fully understood this patient's entire financial and social lived experience.” Then through that, by having that comprehensive intake, you have better mapping onto the financial resources that exist— and what they qualify for.”
		• “It's some kind of robust tracking mechanism that includes dates and schedules of encounters, is a way to document what happens during each encounter, and follow-ups and action items that are needed, and is basically a resource for the navigator to go back to and update and use.”
3. Used patient-specific needs to guide coordination of access to resources	• Using patient needs to direct when meetings are scheduled, which resources are prioritized, etc.	• “I think it was very flexible because in our initial interview with each patient, we would identify what their particular needs were. The intervention allowed us to tailor the assistance we provided to whatever the patient's needs were.”
4. Consisted of strong 1-on-1 relationship between navigators and patients as the cornerstone of financial navigation and the success of the intervention	• 1-on-1, synchronous calls	• “The deep empathetic and authentic relationships that people in those roles [navigators] were able to build with patients going through the cancer experience. It just seemed so crystal clear that patients need that. Patients with financial distress need a person to go to.”
	• In-person sessions	• “It [financial navigation] was much more familiar and personal, and we got a lot of comments on that from patients. That made a huge difference.”
5. Ongoing opportunities exist patients to receive dynamic assistance with applications	• Reviewing current patient needs and circumstances/status of applications at the beginning of each session	• “Because the patients would have regularly scheduled checkups with their navigator to say, “Hey, did you get that paperwork for me that I could turn in for your financial assistance application to the hospital?” Somebody who was doing those regular checks on a very consistent basis, until all of the assistance was completed. I think that's the big difference instead of just handing them a packet and saying, “Here you go. Fill this out, and turn it in.””
		• “But sometimes checking back in with them enabled us to jump on something that needed to be addressed before it became a crisis or before it was too late. Like getting somebody connected to COBRA and COBRA Premium Assistance. That's a very tight timeline. If you're not on top of it, the time limit is passed and then you lose the opportunity for that program.”
		• “I'm going to check in with you in a couple weeks.“ Then it was in the schedule that okay, I need to go check in with Mrs. Jones today. I call Mrs. Jones, and I'm like, ”Hey, were you able to fill that out yourself¿‘ ”Oh, no. I forgot about it.“ Okay, here's a nudge, or ”Oh, yes, except I'm missing the so and so.“ Okay, well maybe I can help you with that.”
6. Actively removed common barriers to accessing resources	• Providing application completion assistance	• “People having trouble gathering documents that they needed to prove their income situation, their health situation, their—eligibility criteria. Helping them with that. Barriers such as they just didn't know about programs. Making sure that we were adept on all of the potential programs depending on their situation and then connecting them with those.“
		• “It doesn't just educate and leverage existing structure, it's really a handholding sort of thing for patients who need it. To actually assist in finishing applications and things like that.”
		• “...this idea of whether certain resources for patients had to be activated by the patient or whether they were activated by providers. We found that many of the resources that were available for patients were patient activated. If patients were not computer savvy or had language barriers or just were shy about asking for resources, they wouldn't necessarily get connected.”
		• “things that we were already well aware of and knew that people needed help with were things like the digital gap. People not having access to online applications. Health and cultural literacy, educational literacy challenges that people had in understanding how to fill out applications.”

**Table 2:** LIFT implementation core functions.

**LIFT implementation core functions**		
**Core function: the “what”—what facilitated LIFT's implementation?**	**Example forms: the “how”—how has LIFT accomplished the core function?**	**Example quotes**
1. Engaged facilities that had the resources necessary to implement FN	• Existing social and financial assistance programs that were *separate* from revenue and mechanisms for screening and referral	• “I think the key—our special sauce, to get to something you said earlier, is that we developed this within the existing system”
2. Developed financial navigators' capability to implement FN	• Training	• “That was really critical to the replicability...anybody with a certain base level of skills could be trained to do this.”
	• Tailored coaching calls	
	• Online peer communication	
3. Provided a comprehensive case management system to enable financial navigators to efficiently and effectively coordinate and track resource access	• Comprehensive intake process and detailed tracking mechanisms	• “It's some kind of robust tracking mechanism that includes dates and schedules of encounters, is a way to document what happens during each encounter, and follow-ups and action items that are needed, and is basically a resource for the navigator to go back to and update and use.”
4. Engaged facilities that were willing to implement FN	• High organizational readiness	• “I think there was a lot of momentum to address this as an interest of the hospital, the health system, that there was—they'd been looking at this and they were wanting to—they were ready to do a pilot, so I think that helped”
5. Supported financial navigators by connecting them with their peers	• Online peer communication	• “having the strategies of the peer support monthly calls and technical assistance”

### Intervention core functions

The change underlying LIFT's effectiveness, represented by LIFT's intervention core functions, can be explained using Transaction Cost Economics theory (24). Transaction Cost Economics proposes that there are costs associated with planning, implementing, and enforcing transactions with other organizations. In the context of FN, transactions include soliciting, coordinating, and administering financial resources and support from the cancer programs and external organizations (e.g., foundations) to address FT. The costs of FN transactions are particularly high due to their specificity, uncertainty, and frequency. Negotiating transactions to garner financial support requires substantial skill. Information regarding what funding was available, when it would be available, and to whom it was available was difficult to ascertain and depended upon irregular information-gathering transactions. Transaction Cost Economics proposes that transaction costs can be minimized with governance structures; LIFT represents a compilation of governance structures that minimized the costs associated with FN. LIFT involved financial navigators with specialized training that allowed them to develop strong one-on-one relationships with patients and use patient-specific needs to guide coordination of access to resources. As an example, one interview participant said, “It just seemed so crystal clear that patients [with financial distress] need[ed]…the deep empathetic and authentic relationships that people in those roles [navigators] were able to build.” Another participant said, “[I]n our initial interview with each patient, we would identify what their particular needs were. The intervention allowed us to tailor the assistance we provided to whatever the patient's needs were.” As a result, LIFT financial navigators reported feeling confident in their roles; the quality, trust and rapport of the navigator-patient relationship; and their ability to remove barriers to reducing FT.

LIFT addressed FN transaction irregularity and uncertainty by offering dynamic assistance to patients. One interview participant said, “…[P]atients would have regularly scheduled checkups with their navigator to say, “Hey, did you get that paperwork for me that I could turn in for your financial assistance application to the hospital?” Somebody who was doing those regular checks on a very consistent basis until all of the assistance was completed. I think that's the big difference instead of just handing them a packet and saying, “Here you go. Fill this out, and turn it in.”” This dynamic patient assistance was supported by systematic methods of cataloging FN resources and tracking patient information, allowing financial navigators to efficiently remove common barriers to accessing resources. An interview participant said, “Once identified, having a comprehensive intake assessment that wasn't just, “Tell me about your job,” or, “Tell me about your insurance status,” but that was driven by—almost like a checklist of, “Okay, well, let me just go through and make sure that I've fully understood this patient's entire financial and social lived experience.” Then through that, by having that comprehensive intake, you have better mapping onto the financial resources that exist— and what they qualify for.” Another participant said, “It was like harnessing knowledge that existed but wasn't always systematized in a way that—every encounter might've been a little bit different, and pulling all that information together and coordinating it and then, yeah, like you said, structuring, organizing it, and helping improve the process so that the process itself didn't rely on one person's institutional knowledge.” Without systematic methods of cataloging FN resources and tracking patient information, financial navigators were previously reliant on personal knowledge, paper-based notes, and less standardized approaches to meeting patients' needs.

### Implementation core functions

LIFT's implementation core functions can be explained using Organizational Readiness Theory (25). Organizational readiness is defined as organizational members' shared resolve and perceived capability to implement a change such as LIFT. Consistent with Organizational Readiness Theory, we found that LIFT's implementation required engaging facilities that were willing to do the work of implementation and had the resources required to implement LIFT. One interview participant said, “I think there was a lot of momentum to address this as an interest of the hospital, the health system, that there was—they'd been looking at this and they were wanting to…so I think that helped.” Another said, “I think the key—our special sauce…is that we developed this within the existing system.” Implementing LIFT also required developing financial navigators' capability and providing the systems necessary to support implementation. One participant said, “That was really critical to the replicability...anybody with a certain base level of skills could be trained to do this.”

## Discussion

We identified core functions associated with the LIFT FN intervention and its implementation and mapped them onto existing theories of change. Our findings suggest that FN reduces FT by minimizing the transaction costs associated with delivering financial support to patients experiencing FT. Intervention core functions minimizing transaction costs included systematically cataloging knowledge and tracking patient-specific information related to eligibility criteria for FT relief. Furthermore, repeat contacts between the financial navigator and participant created an ongoing relationship, removing common barriers to accessing resources. We also found that the successful implementation of LIFT was predicated on organizational readiness. Implementation core functions included having engaged sites with the resources and willingness necessary to implement FN. Developing navigators' capabilities to implement LIFT—through training, an established case management system, and connections to peer navigators—were also identified as implementation core functions. These findings serve to clarify features of the LIFT intervention that must be retained when adapting LIFT to new settings and populations. They also serve to supplement studies evaluating financial navigation interventions more broadly by providing a framework of core functions to be built upon, revised, and adapted over time (26).

The success of LIFT and other FN interventions is predicated on retention of core functions, while being attentive to the local, contextually-specific needs that may warrant adaptation to improve fit, for example, in patients living in rural areas and seeking care at community cancer centers. Cancer care in rural settings is unique (27); resource constraints and limited geographic access to care providers may directly affect how FN is delivered (28) (for example, in terms of the financing and differences in workflows and duties of rural navigators, as well as the delivery of FN *via* phone or in person). Our findings suggest that LIFT can reduce FT in even the most challenging practice contexts as long as its core functions are retained. Our findings, taken with previous adaptation studies for which organization theories were also relevant explanations of intervention and implementation core functions, suggest that organization theories are particularly relevant for explaining how and why interventions such as LIFT are adopted, implemented, and sustained in new contexts (29).

Financial hardship associated with cancer is prevalent, yet interventions to alleviate its burden are few and far between. FN holds considerable promise, but like most patient navigation models that respond to patient-centered needs and contextual realities, the details of these interventions can be perceived as amorphous. Our findings provide generalizable guidelines for those seeking to implement FN. Although FN generally, and LIFT specifically, may take on specific forms in a particular context or population, the success of such programs is buoyed by its core intervention and implementation functions. LIFT's core functions thus enable diverse cancer care settings to implement programs that are expected to succeed for various populations.

Our study has limitations. Our approach to identifying core functions was not experimental; that is, we do not have data regarding the causal relationship between the core functions that we identified and key implementation and intervention outcomes. However, our approach is theory-based and thus posits specific mechanisms underlying the relationship between core functions and key implementation and intervention outcomes. These relationships should be studied in future work. We also relied on the perspectives of LIFT designers and implementers. To the extent that existing study documents and interview data reflect a biased view of LIFT, the core functions that we identified may be inaccurate. However, we included a diverse group of LIFT designers and implementers, ranging from study principal investigators to financial navigators to research assistants, and relied on extensive intervention documents to promote as accurate a representation of the intervention and its implementation as possible. This study also reflects an intervention developed and delivered initially in a single, large, academic medical center and may not be representative of other FN interventions that are concurrently being developed and tested.

Despite these limitations, the strength of this study lies in its theory-based approach to identifying an initial set of core functions underlying FN interventions, using LIFT as a case example. The core functions that we identified in this study should be regarded as living documents to be continuously revised based on the implementation of LIFT in new contexts and populations.

In conclusion, to our knowledge, this is the first study to report core functions of a FN intervention. Our findings contribute to a growing body of literature that reports intervention and implementation core functions (30–32). Identifying core functions is critical for extending the benefits of effective interventions into new contexts and populations. Indeed, we expect findings from this study to improve the practice of FN for patients with cancer because it will demonstrate the benefits of a scalable, pragmatic, individualized intervention that focuses on patients at high risk of FT. It also can be delivered remotely outside the clinical encounter, equipping oncology practices, health systems and payers with a process to optimize cancer care delivery through addressing FT.

## Data availability statement

Data from this study will be available upon reasonable request to the corresponding author.

## Author contributions

SW, SB, and DR conceived of and designed the study, oversaw all data collection, analyses and interpretation, wrote/edited and approved the final manuscript. CW, MM, MG, NP, CB, CR, and JR contributed data to the manuscript, revised and approved the final manuscript. CS and RB aided in interpretation of the data, revised and approved the final manuscript. All authors agree that they are accountable for all aspects of the work.

## Funding

This research was supported by the National Cancer Institute (NCI) at the National Institutes of Health (NIH) via three grants: 1-R01-CA240092-02 (SW and DR), 3-P30-CA016086-44-S4, PI: Earp, Project Leads: SW and DR), and P30CA012197-45S5 (Wake Forest's Supplement, PI: Boris Pasche, Project Leads: RB and SB). Additional funding for this project was provided by the National Comprehensive Cancer Network and Pfizer Independent Grants for Learning and Change (PI: SW and DR) and the University of North Carolina Innovation Center. In addition, SW, SB, and CW receive support for this work, in part, through the Cancer Prevention and Control Research Network, funded by the Centers for Disease Control and Prevention of the U.S. Department of Health and Human Services (HHS) (U48 DP006400). CB is supported by a Cancer Care Quality Predoctoral Traineeship, UNC-CH, Grant No. T32-CA-116339. SW receives grant funding paid to her institution from Pfizer Foundation.

## Conflict of interest

The authors declare that the research was conducted in the absence of any commercial or financial relationships that could be construed as a potential conflict of interest.

## Publisher's note

All claims expressed in this article are solely those of the authors and do not necessarily represent those of their affiliated organizations, or those of the publisher, the editors and the reviewers. Any product that may be evaluated in this article, or claim that may be made by its manufacturer, is not guaranteed or endorsed by the publisher.

## Author disclaimer

The contents are those of the author(s) and do not necessarily represent the official views of, nor an endorsement, by CDC/HHS, or the U.S. Government.
