# Supplementary material for: Core functions of a financial navigation intervention: An in-depth assessment of the Lessening the Impact of Financial Toxicity (LIFT) intervention to inform adaptation and scale-up in diverse oncology care settings
Source: Front Health Serv. 2022 Nov 9;2:958831. doi: 10.3389/frhs.2022.958831 (PMC10012722; doi:10.3389/frhs.2022.958831)
Supplement: Supplementary file 1 [file Data_Sheet_1.DOCX]

**Core Functions Cheat Sheet: LIFT**

**What:** LIFT

- LIFT is a program that enlists trained Financial Navigators to assist cancer patients in relieving the financial burden of their care by identifying and accessing resources to reduce cancer-related financial distress. To qualify for the program, patients must be over the age of 18 with any type and stage of cancer and a confirmed cancer diagnosis within 5 years or be a patient living with advanced disease, score 22 or lower on the COST measure indicating high financial distress, and patients must be able to read and speak English.

**Where:**

- Piloted at UNC Chapel Hill
- Initial recruitment usually took place while a patient was already meeting with a social worker
- Enrollment and completion of the intake form took place in person or over the phone, depending on their patient’s availability and needed documentation
- In person recruitment took place at the hospital

**When:** Recruitment took place when patients were present at the hospital and had a meeting with a social worker/financial navigator. Follow-ups occurred on a flexible bi-weekly basis either in person or by phone.

**Why:**

- Financial toxicity (FT): the financial burden that healthcare places on patients and their families
- FT is a major hardship for cancer patients
- Out-of-pocket direct medical costs (e.g., copayments, coinsurance, over-the-counter and prescription drug costs, medical supplies) and out-of-pocket indirect and non-medical costs (e.g., transportation, lost wages, caregiving expenses) increase the burden
- One in three Americans experience significant financial burden as a result of medical care.
  - High out-of-pocket healthcare costs is greater for cancer patients than for patients with other chronic illnesses
  - Disproportionately burdensome in rural communities
- Health insurance does not fully protect against financial hardship associated with cancer.
  - Financial Navigation is needed to assist patients and caregivers with identifying and accessing resources to reduce cancer-related financial distress.
- There is need to enlist trained financial navigators to prevent and mitigate cancer-related FT

**How (does it produce change)**:

- Provides a navigator to assist patients with the complicated process of finances during cancer treatment.
- LIFT leverages existing structures and processes that your center already has in place to support patients
- It builds capacity and improves quality of care, while reducing duplication of effort and integrating workflows
- LIFT educates patients about programs and services that may help address their financial distress
- LIFT assists patients in applying for, and receiving the benefits from, existing programs and services such as disability, subsidized insurance coverage and SNAP benefits.
