# Supplementary material for: Core functions of a financial navigation intervention: An in-depth assessment of the Lessening the Impact of Financial Toxicity (LIFT) intervention to inform adaptation and scale-up in diverse oncology care settings
Source: Front Health Serv. 2022 Nov 9;2:958831. doi: 10.3389/frhs.2022.958831 (PMC10012722; doi:10.3389/frhs.2022.958831)
Supplement: Supplementary file 2 [file Data_Sheet_2.DOCX]

**Interview guide for identifying core functions of LIFT**

*Thank you for agreeing to participate in the interview today. This interview is part of a larger research study I am conducting to adapt the LIFT intervention originally developed by research colleagues at UNC Lineberger Cancer Center to evaluate the implementation and effectiveness of a Financial Navigation intervention through the use of a Financial Patient Navigator (FPN) for FUND study patients.*

*The first phase in my research is to learn more about the original intervention. Specifically, I want to: 1) identify the key activities of the original intervention 2) identify ‘core functions’ of LIFT (*the essential principles and intervention activities necessary to produce desired outcomes) 3) *identify any lessons learned from the original intervention. Since you were a member of the study team involved in the original intervention, I wanted to speak to you to hear your perspective on these topics. I’m then going to use what I learn in these interviews to inform how I adapt the intervention.*

*Our conversation today should last about 30-60 minutes. Anything you say here today will be kept confidential, meaning anything you say during this interview will not be attributed to you specifically. My colleagues are on the call to take notes; to fill in any gaps in our notes later.* ***Do you have any questions for us? we’d also like to record our call today, is that OK with you?***

*[Based on response, start recording]*

**1. Participant Information**

- **Question**:
  1. Could you describe your role on the study team that developed the original intervention?

**2.List of EBI activities**

*Prior to this interview, I provided you with a ‘cheat sheet’ that we put together describing the activities of the original intervention. We developed this ‘cheat sheet’ along with a process flow diagram based on details of the intervention that were described in the LIFT Standard Operating Procedure. Would you please take a few moments – no rush – to review the documents?* [Provide ~3-5 mins to review the sheet, if they have not yet reviewed it.]

*I’d like to confirm some details of the original intervention with you – knowing what activities were done in the original intervention will help me adapt the intervention to the new setting.*

- 1. How appropriately does the cheat sheet describe the original intervention?
     - Probe: Do you see anything missing? Any gaps in activities you’d like to fill in?
  2. How formalized was the protocol (were there deviations from it)?
  3. Does this list reflect your experience with LIFT activities? Would you omit or add any?

**3. Theoretical level of change, causal pathways**

- 1. Can you talk about the rationale for what the intervention was designed to do?
  2. Based on your experience, can you describe barriers to financial navigation you encounter in practice? / What were the barriers to financial navigation that motivated the intervention?
  3. Which barriers to financial navigation was LIFT designed to address? / How is LIFT intended to address the barriers you just described (if at all)? [Make sure respondent describes *how* LIFT addressed barriers to change, not just which activities.]

**4. Core Functions of the Intervention**

*Now we’re going to be shifting from more straightforward ideas about what the intervention is to thinking more about which features you think made the intervention work.*

- 1. Which LIFT activities contributed most to addressing financial toxicity?
     - a. Probe: What about the activity was essential – e.g., the person conducting the activity; mode of activity (in-person vs written)?
  2. From your perspective, what aspects of these key activities are essential to the success of LIFT? / Which LIFT activities/principles would you maintain at all costs?
     - Probe: what about it was essential – e.g., who is conducting the activity; mode of activity (in-person vs written)?
  3. Which LIFT activities/principles seem less essential, and could be cut out or modified?

**Part 2: Brainstorming potential differences between LIFT and FUND**

*We are now going to talk about unique features of the LIFT target population and UNC. This will help us to learn which unique features will we need to consider as we adapt LIFT for our purposes with FUND.* ***Is there anything about LIFT’s patient population, care setting and delivery, policy and external environment, or attitudes about financial navigation that you think might have made LIFT uniquely successful or perhaps limited its effectiveness?*** [treat everything after this as probes]

**1. Patient Population**

- **Goals of care:**
  - What did UNC seek to achieve with this intervention?
  - How do you think goals of care may differ between English- and Spanish-speaking patients?
  - How may goals of care differ for patients who score greater than or equal to 23 on the COST measure?
- **Clinical condition:**
  - How may the clinical status of LIFT patients impact the intervention?
    - This could cover differences in terms of functional and cognitive status, as well as other clinical factors, like risk of hospitalization or likelihood of death within 1 year.
  - What about patients who score 23 or greater on the COST measure?
- **Demographics:**
  - What are the key demographics of the UNC LIFT patient population?
    - factors like age, primary payer, etc.
  - How may the demographics between English-speaking and Spanish-speaking patients different?
  - What about patients who score 23 or greater on the COST measure?
- **Symptom/service needs:**
  - How may the symptom and service needs of LIFT patients at UNC differ from other communities?
    - This would cover differences (or similarities) in terms of symptom (e.g., pain, shortness of breath) and service needs (need of an aide, social worker, clergy, transportation, etc.). This could include physical as well as psychosocial symptoms.
  - How may the symptom and service needs of English-speaking patients compare to Spanish-speaking patients?
  - What about patients who score 23 or greater on the COST measure?
- **Social Factors:**
  - What do believe are some of the major differences in social factors between LIFT patients at UNC and patients in other communities?
    - This would cover differences (or similarities) in terms of social factors, such as the presence/role of a family caregiver.
  - What may be some of the major differences in social factors between English- and Spanish-speaking patients? .
  - What about patients who score 23 or greater on the COST measure?
- **Preferences**:
  - What may be some of the major differences between care preferences for LIFT patients at UNC and patients in other communities?
  - What do you think may be a major difference in preference between English- and Spanish-speaking patients?
  - What about patients who score 23 or greater on the COST measure?

**2. Care Setting and Delivery**

- **Care delivery model:** How does care delivery for UNC and LIFT vary from other communities? This would include:
  - Who is delivering care (nurses vs MD, what is the role of the MD in care delivery? What is the role of community physicians/clinicians vs LIFTclinicians?)
  - What are the major differences in care setting and delivery between English- and Spanish-speaking patients? This would cover differences (or similarities) in terms of setting and delivery.
  - What about patients who score 23 or greater on the COST measure?
  - Main types of services provided?
  - How often services are provided?
  - Where services are provided?
- **Referral sources:** What are the primary referral sources for each care setting (e.g., financial assistance)? What are the triggers for referral (e.g., acute event)?
  - English- vs Spanish-speaking?
  - Less than 23 on COST vs 23 or greater?
- **Agency: Based on your experience at UNC and what you know about Wake Forest Baptist Health, can you think about** any major differences there may be in terms of how these systems function to implement a program like LIFT?

**3. Policy and External Environment Context**

- **Regulatory requirements/larger policy context**: **Based on your experience at UNC and what you know about Wake Forest Baptist Health, can you think about** any major differences between the larger policy context or external environment for LIFT vs FUND agencies (e.g., differences in Commission on Cancer requirements, relationship with financial assistance organizations, insurers)?

**4. Financial Navigation**

- **Attitudes and knowledge about financial navigation**: How may attitudes and knowledge about financial navigation different between UNC’s LIFT and Wake’s FUND from the perspective of:
  - Patients?
  - Staff?
- **Relationship with financial toxicity once patient is enrolled:** How does the relationship between the patient and the navigator(?) differ once the patient is enrolled in financial navigation?

**Those were the only questions we had planned for today. Is there anything else you think I might want to know that we haven’t covered?**
